# Engineering Nano-Antibiotics for Accelerating Wound Healing in Drug-Resistant Bacterial Infections

**DOI:** 10.3390/molecules31111957

**Published:** 2026-06-04

**Authors:** Wenmin Yan, Zihao Shen, Shilan Liang, Chaozhong Li, Guangwei Feng, Jinming Zhu, Jian Feng

**Affiliations:** 1 School of Basic Medical Sciences, Guizhou Medical University, Guiyang 561113, China; qweryan1233@163.com (W.Y.); zihaoshengmu@163.com (Z.S.); sllianggmu@163.com (S.L.); lichaozhong@gmc.edu.cn (C.L.); 2 Guizhou Provincial Health and Development Research Center, School of Medical Humanities, Guizhou Medical University, Guiyang 550025, China

**Keywords:** bacterial infections, wound healing, nano-antibiotic, nanoplatelets

## Abstract

Plenty of nano-antimicrobial materials have been developed successively, aiming to address severe clinical challenges such as wound healing disorders and high postoperative mortality rates caused by drug-resistant bacterial infections. However, their reliance on external stimuli (light, thermal energy, or exogenous H_2_O_2_ addition) for bactericidal activation severely hampers clinical translation from bench to bedside. Herein, we report an engineered Cu/CeO_2_ nanoplatelet (NP) system that functions as a stimulus-independent, time-dependent nano-antibiotic against methicillin-resistant *Staphylococcus aureus* (*MRSA*), while also exhibiting broad-spectrum antibacterial activity. In a skin wound model infected with *MRSA*, topical application of only 1 μg/mL achieved near-complete wound closure within 10 days. The satisfactory therapeutic effect is concluded: (1) Cu/CeO_2_ NPs continuously release Cu^2+^, which damages the integrity of bacterial cell membranes and achieves efficient sterilization. (2) The antioxidant stress capacity, peroxidase, and catalase-like activity effectively alleviate oxidative stress and hypoxia conditions in the infected microenvironment, and synergistically exert multiple biological effects such as anti-inflammatory, promoting collagen deposition and the formation of new blood vessels. This study not only provides a feasible pathway for the clinical application of antibacterial nano-materials, but also offers theoretical support and practical examples for the rational design of multifunctional nano-antibiotics.

## 1. Introduction

In recent years, the research on responsive antibacterial materials has witnessed an explosive growth, with its core application direction focusing on the intervention of bacterial infection-induced skin wounds, especially in addressing chronic and hard-to-heal wound infections caused by drug-resistant bacteria such as methicillin-resistant *Staphylococcus aureus* (*MRSA*) [1,2,3,4]. Most of the reported materials typically require near-infrared light response or H_2_O_2_ response, generating active radicals through photothermal conversion effect or Fenton reaction to achieve bacterial killing [5,6,7,8,9,10,11]. However, the repair of damaged skin wounds is a long-term, dynamic and highly ordered biological process, involving multiple key steps such as continuous antibacterial action, redox homeostasis and inflammation regulation, cell proliferation, and skin remodeling [12,13]. During the healing cycle, the wound is usually covered by drugs or scabs and remains in a state of no light and hypoxia for a long time [14,15]. Relying solely on the short and transient bactericidal window provided by near-infrared light irradiation or H_2_O_2_ addition makes it difficult to coordinate and regulate the above multiple biological functions in the temporal and spatial dimensions [16,17,18]. Responsive antibacterial materials cannot be used as substitutes for antibiotics, and their clinical application and transformation are greatly limited. Although further processing the materials into biomedical devices such as gel dressings can enhance their practical application performance and accelerate the transformation process, the optimization of the intrinsic properties of the materials remains the core foundation of research and development.

In view of the multiple pathological features of drug-resistant bacterial skin wounds, including persistent bacterial clearance, uncontrolled inflammatory responses, and delayed repair caused by local tissue hypoxia after drug administration, the ideal antibiotic alternative with multiple functional synergies should possess the following core capabilities: (1) efficiently kill drug-resistant bacteria without relying on external stimuli; (2) dynamically clear excessive reactive oxygen species to alleviate oxidative stress damage and regulate the orderly inflammatory microenvironment; (3) provide oxygen in situ to improve the hypoxic state of the wound, supporting cell metabolism and tissue regeneration [12,13,14,15].

Due to its excellent biocompatibility, antioxidant stress capacity, and peroxidase (POD) and catalase activity (CAT), CeO_2_ have been widely used in the intervention research of inflammation-related diseases [19,20]. The consecutive enzyme activity of CeO_2_ can effectively catalyze ROS in the inflammatory environment into O_2_, thereby improving the local hypoxia state of the wound and promoting tissue repair. However, CeO_2_ themselves do not have antibacterial properties [21]. Currently, constructing composite materials with CeO_2_ carriers has become the mainstream strategy for dealing with inflammatory infectious diseases. Cu^2+^ can cause the denaturation of proteins in bacterial biofilms by interacting with the thiol groups on the biofilm surface, leading to the imbalance of the biofilm’s homeostasis and its rupture, thus achieving sterilization [22,23,24]. However, high concentrations of Cu^2+^ have significant cupro-Fenton reaction activity and can induce cuproptosis. Therefore, in the design and application of a Cu-based antibiotic alternative, it is crucial to regulate the loading amount of Cu and its bioavailability [24,25].

In this study, a series of Cu/CeO_2_ nanoplatelets (NPs) with different Cu contents was designed and synthesized. The loading of Cu nanoparticles endows Cu/CeO_2_ NPs with the ability to spontaneously release Cu ions without external stimulation, achieving continuous and efficient killing of *MRSA* by destroying the bacterial cell membrane. Notably, the antibacterial mechanism of Cu/CeO_2_ NPs is significantly different from that of the reported similar antibacterial materials that rely on the Fenton reaction [26]. The Cu/CeO_2_ NPs reported in this work achieve spontaneous sterilization, anti-inflammation, and oxygen supplementation synergistic performance, providing a new pathway to break through the limitations of traditional antibacterial materials and promoting the engineering application of nano-antibiotics in clinical practice.

## 2. Results and Discussion

**Figure 1:**
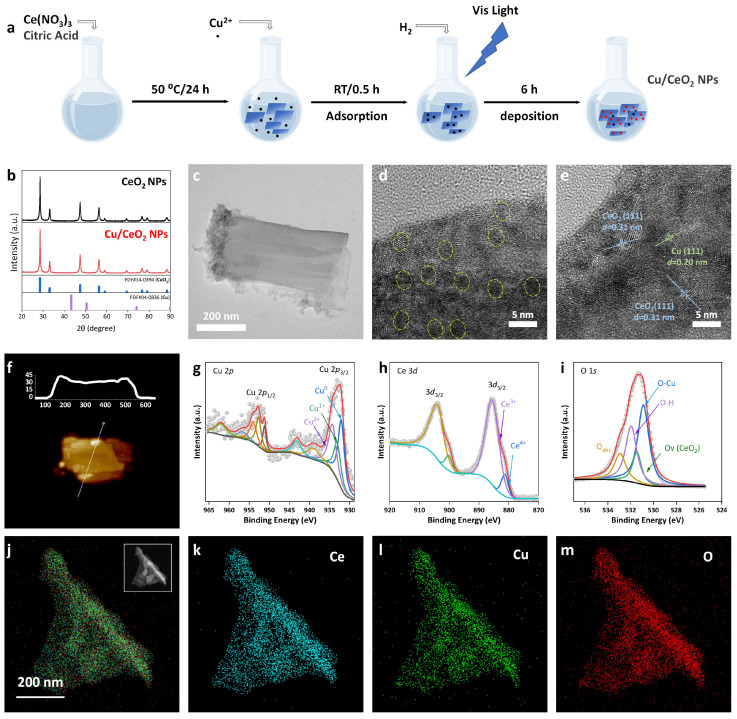
Characterization of Cu/CeO_2_ NPs. (**a**) Schematic drawing of synthesizing procedures. (**b**) XRD pattern of Cu/CeO_2_ NPs and CeO_2_ NPs. (**c**) LRTEM image. (**d**) HRTEM image, with yellow circles presenting defects. (**e**) HRTEM image, with the annotation of lattice spacings. (**f**) AFM images of Cu/CeO_2_ NPs. XPS spectra of Cu/CeO_2_ NPs with elements of Cu (**g**), Ce (**h**), and O (**i**). EDS-mapping image of Cu/CeO_2_ NPs with elements of merged (**j**), Ce (**k**), Cu (**l**), and O (**m**).

**Figure 2:**
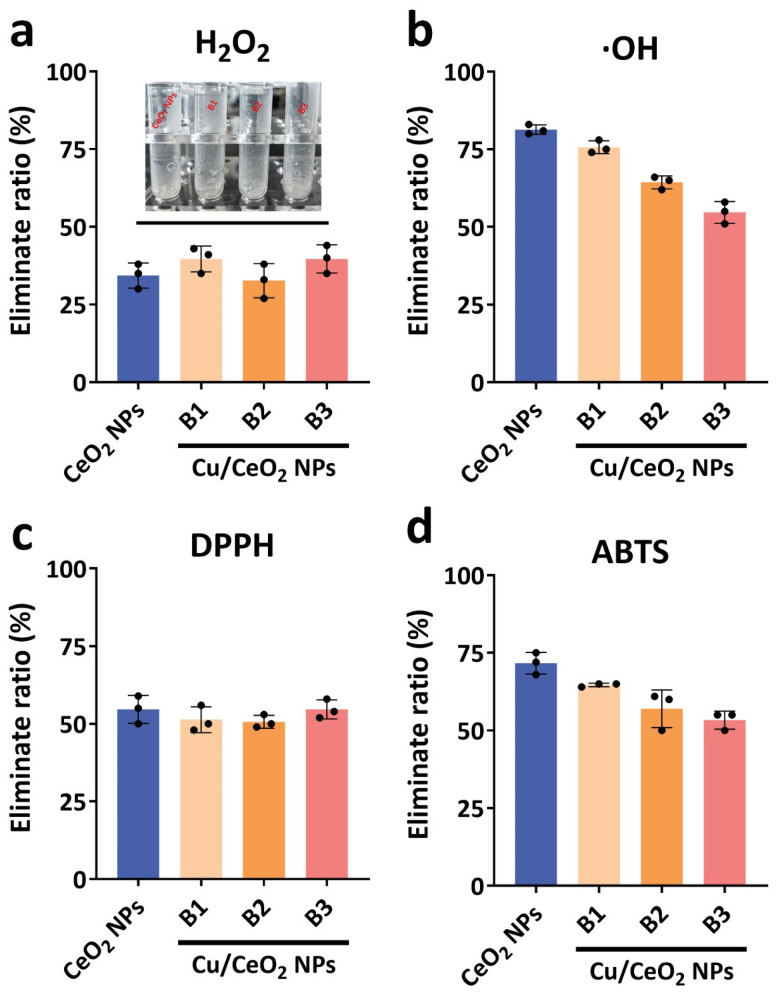
In vitro antioxidant stress performance test. (**a**) CAT enzyme activity. (**b**) Eliminate ratio of materials for (**b**) ·OH, (**c**) DPPH, and (**d**) ABTS radical.

**Figure 3:**
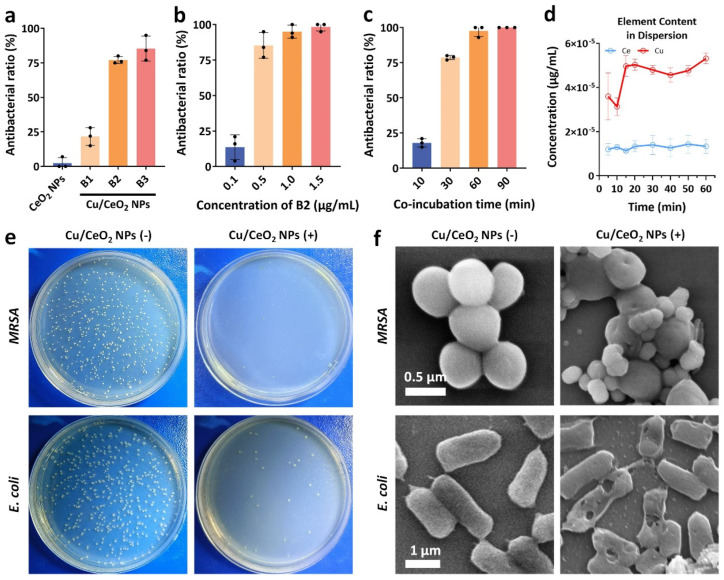
In vitro antibacterial performance test. (**a**) Antibacterial ratio comparison of CeO_2_ NPs, B1, B2, and B3. (**b**) Concentration independence antibacterial ratio of B2. (**c**) Co-incubation time independence antibacterial ratio of B2 at 0.5 μg/mL. (**d**) Cu and Ce element content of ·Cu and Ce in dispersion. (**e**) Representative images of culture media (for *MRSA* and *E. coli*) Cu/CeO_2_ NPs intervened via Cu/CeO_2_ NPs. (**f**) SEM images of bacteria (for *MRSA* and *E. coli*) intervened via Cu/CeO_2_ NPs.

**Figure 4:**
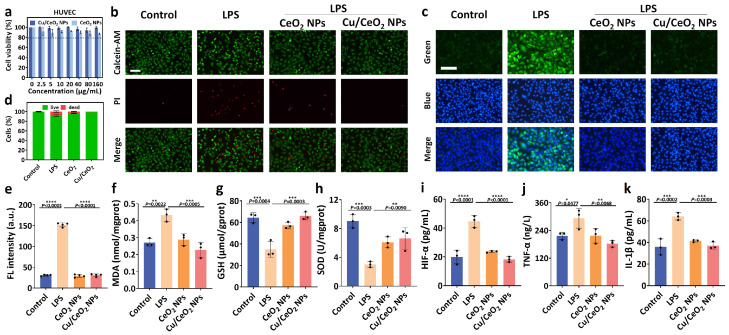
In vitro cell performance of Cu/CeO_2_ NPs. (**a**) Cytotoxicity of the Cu/CeO_2_ NPs with different concentrations to HUVEC. Images of living dead cell staining (**b**) and corresponding statistical results (**d**), scale bar = 200 μm. Images of intracellular ROS staining (**c**) and corresponding statistical results (**e**), scale bar = 50 μm. Express level of intracellular MDA (**f**), GSH (**g**), SOD (**h**), HIF-α (**i**), TNF-α (**j**), and IL-1β (**k**).

**Figure 5:**
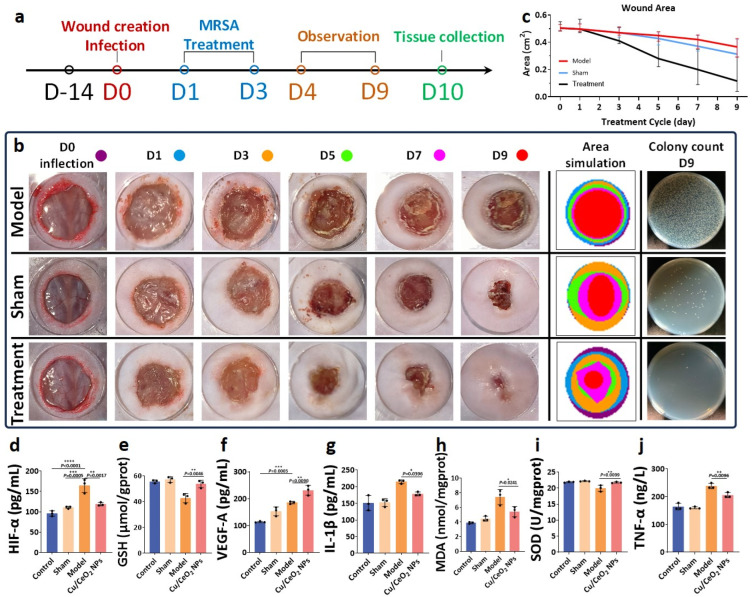
The effect of Cu/CeO_2_ NPs on wound healing in *MRSA*-infected mice model. (**a**) Schematic drawing of modeling and treatment period. (**b**) Representative images of the wound (**left panel**), area simulation (**middle panel**), and bacterial statistics (**right panel**) of the wound on the 9th day. (**c**) Statistical analysis of wound area in different groups of mice (*n* = 6). Express level of MDA (**d**), GSH (**e**), SOD (**f**), TNF-α (**g**), IL-1β (**h**), HIF-α (**i**), and VEGF-A (**j**) in the serum.

**Figure 6:**
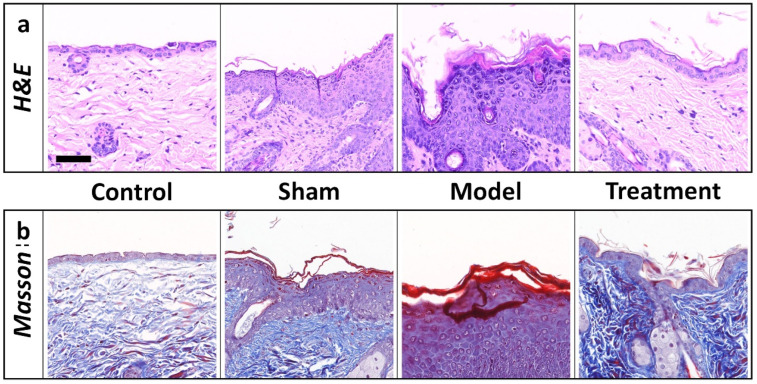
The H&E (**a**) and Masson (**b**) staining of skin sections from mice in different groups.

Figure 1 reports the synthesis and characterization of the material, Figure 2 reports the in vitro chemical reductivity of the material, Figure 3 reports the in vitro antibacterial performance of the material, and Figure 4 reports the antioxidant stress capacity of the material at the cellular level. With these prerequisites in place, we applied the material to the treatment of *MRSA*-infected mouse wounds, and the related results are reported in Figure 5 and Figure 6.

### 2.1. Synthesis and Characterization of Cu/CeO_2_ NPs

The synthetic process of the materials is schematically drawn (Figure 1a). CeO_2_ NPs were prepared by a reported synthesis method [27]. Subsequently, Cu^2+^ were adsorbed onto CeO_2_ NPs in solution, and Cu^2+^ was reduced and anchored on the surface of CeO_2_ NPs in the form of nanoparticles under a hydrogen atmosphere by photo-deposition to prepare Cu/CeO_2_ NPs [28]. According to the different concentrations of Cu precursors added, we obtained Cu/CeO_2_ NPs with low, medium, and high Cu loading, which were named Batches 1 (B1), 2, and 3, respectively, and corresponding Cu/Ce mass ratios were presented (Figure S1).

The structural characterization results of Cu/CeO_2_ NPs represented by sample B2 are also presented in Figure 1. X-ray diffraction (XRD) analysis (Figure 1b) indicates that the diffraction pattern of samples can be well matched with the standard card of CeO_2_. In the diffraction peaks of Cu/CeO_2_ NPs, no characteristic diffraction peaks of face-centered cubic structure metallic Cu were observed, suggesting that Cu did not form large particles on the material surface. XRD patterns of aged samples in air show significantly weakened and broadened characteristic peaks for CeO_2_ and Cu/CeO_2_ NPs, confirming surface adsorption of environmental water and subsequent crystallization (Figure S7). Low-resolution transmission electron microscopy (LRTEM) observations (Figure 1c) reveal that Cu/CeO_2_ NPs exhibit a layered stacking microstructure. High-resolution (HR) TEM images (Figure 1d) further reveal a large number of structural defects (yellow circles) such as lattice distortions, grain boundaries, and dislocations in the material. The lattice spacings (Figure 1e) of 0.31 nm and 0.20 nm correspond to (111). Atomic force microscopy (AFM) analysis (Figure 1f) indicates that the material forms a relatively flat two-dimensional nanosheet structure on the substrate, with a thickness of approximately 40 nm. Dynamic light scattering (DLS) (Figure S2) and zeta potential test (Figure S3) results show that Cu/CeO_2_ NPs exhibit around 1100 nm hydrodynamic size and 12.9 mV zeta potential. The zeta potential of Cu/CeO_2_ NPs is significantly higher than that of CeO_2_ NPs, indicating that Cu species are enriched on the material surface in the form of cations. X-ray photoelectron spectroscopy (XPS) analysis of Cu elements (Figure 1g) shows that Cu species coexist in three chemical states in the sample: metallic Cu (932.5 eV), Cu^+^ (933.5 eV), and Cu^2+^ (935.0 eV) [29,30]. This conclusion is consistent with the DLS results. The Ce spectrum (Figure 1h) indicates that Ce mainly exists in the form of Ce^3+^ (886.0 eV), with a small amount of Ce^4+^ (881.7 eV), confirming the presence of significant Ce^3+^/Ce^4+^ mixed valence states in the material [31].

The binding energy at 530.9, 531.5, 532.0, and 532.9 eV in the O spectrum (Figure 1i) is attributed to O-Cu, Ov (surface of CeO_2_), O-H, and absorbed O, respectively [32,33]. The whole XPS spectrum was also presented (Figure S4). Energy dispersive X-ray spectroscopy mapping (EDS-mapping) results (Figure 1j–m) show that Cu elements are highly uniformly distributed on the CeO_2_ carrier. Cu/CeO_2_ NPs were successfully synthesized. The material has typical two-dimensional sheet-like structural features, with Cu uniformly dispersed on the surface of the CeO_2_ carrier, resulting in a rich number of active sites on the material surface. In the material, Cu exists in both metallic and ionic forms. The introduction of Cu species promotes the generation of Ce^3+^, thereby effectively enhancing the oxygen vacancy concentration and surface reduction ability of the material, providing a certain structural basis for application in antioxidant stress.

To systematically evaluate the reproducibility of the synthesis process of Cu/CeO_2_ NPs, three batches of Cu/CeO_2_ NPs were independently prepared and characterized by their hydrated particle size and zeta potential (Figure S4). The average hydrated particle size of the three batches was 1103 ± 64 nm, and the zeta potential was −21.6 ± 2.4 mV, with inter-batch differences within the reasonable experimental error range. These results confirm that the synthesis reported strategy has good process robustness and batch consistency. To ensure the reliability of the properties, subsequent evaluations of chemical reduction activity and in vitro antibacterial performance were all based on these three batches of repeatedly synthesized samples.

### 2.2. In Vitro Chemical Reductivity and Enzymatic Activity

The CAT enzyme activity and in vitro chemical reduction test results of the materials are shown in Figure 2, with CeO_2_ NPs as the control group. The experimental details are provided in the Enzyme Activity and Chemical Reduction Test Section. The inset of Figure 2a shows that a large number of bubbles appeared during the reaction of the four materials with H_2_O_2_ solution, which visually confirmed their CAT enzyme activity. Quantitative analysis indicated that B1, B2, B3, and CeO_2_ NPs all exhibited significant scavenging effects on H_2_O_2_, although no differences were observed among the groups. However, the standard deviation within each test group was relatively large, which was speculated to be caused by inaccurate absorbance measurement due to the generation of O_2_. In addition, B2 Cu/CeO_2_ NPs exhibited significantly enhanced POD and SOD enzyme activities compared to CeO_2_ NPs (Figure S5). As for ·OH, DPPH radicals, and ABTS radicals, B1, B2, B3, and CeO_2_ NPs, concentrated at 1 μg/mL, all demonstrated similar scavenging capabilities, suggesting that the loading of Cu did not significantly affect the intrinsic excellent antioxidant stress resistance and enzyme activity of the base material CeO_2_ NPs. The H_2_O_2_ eliminate ratio depended on the ·OH eliminate capacity and CAT of materials. As shown in Figure 2b, the eliminate ratio of ·OH radicals increases with the increase in Cu content. To further evaluate the CAT, the O2 generation rate of the materials was measured using a dissolved oxygen meter (Figure S5). The results indicated that within the same reaction time, the O2 generation decreased with the increase in Cu content, confirming a downward trend in its CAT. Despite the weakened CAT, the overall removal efficiency of H_2_O_2_ by Cu/CeO2 NPs remained stable, suggesting that the enhanced ·OH eliminate capacity effectively compensated for the decline in their CAT.

To ensure proper performance in engineering applications, the ABTS radical scavenging capacity of three batches of Cu/CeO_2_ NPs under uniform experimental conditions was examined (Figure S5). Within a 10 min co-incubation period, 1 μg/mL of Cu/CeO_2_ NPs achieved an average scavenging rate of 61.0 ± 3.8% for the ABTS radical, with an initial absorbance of 1.5 at 732 nm. This highly consistent antioxidant activity demonstrates that the samples from different batches are equivalent in terms of their chemical reduction functionality.

### 2.3. In Vitro Antibacterial Performance

The in vitro antibacterial test results of the materials are shown in Figure 3. In vitro antibacterial experiments were conducted in a co-culture system of bacteria and materials. *MRSA* was used as the Gram-positive bacterial model and *Escherichia coli* (*E. coli*) as the Gram-negative bacterial model. In all co-incubation systems, the bacterial concentration was no less than 1 × 10^8^ CFU/mL under the inoculation conditions. The experimental details are reported in the in vitro antibacterial test section. Figure 3a indicates that under the co-incubation conditions of 0.5 μg/mL and 0.5 h, CeO_2_ NPs have no antibacterial activity, and the antibacterial performance of B1 to B3 increases with the increase in Cu loading, which is in line with the design expectation. B2 was selected as the representative sample for further dose-dependent and time-dependent antibacterial evaluation. Figure 3b shows that under the co-incubation condition of 0.5 h, the inhibition rate of B2 increases with the increase in concentration, and the minimal bactericidal concentration (MBC) value is approximately 1.5 μg/mL. Figure 3c shows that at a concentration of 0.5 μg/mL, the inhibition rate of B2 increases with the increase in co-incubation time with bacteria, and the inhibition rate reaches 100% after 60 min. The representative culture medium images of *MRSA* (Figure 3d) and *E. coli* (Figure 3e) treated with B2 indicate that Cu/CeO_2_ has broad-spectrum antibacterial activity and can be applied to complex wound infection scenarios with multiple coexisting microorganisms. The bacterial morphology images (Figure 3f) show that B2 treatment leads to cell rupture and severe damage to membrane integrity of *MRSA* and *E. coli*, indicating that it mainly achieves efficient bactericidal effect by destroying the bacterial membrane. To further verify the contribution of Cu^2+^ release to the antibacterial effect, the content of the Cu element in the material dispersion was quantitatively determined by inductively coupled plasma optical emission spectrometry (ICP-OES), and the content of the Ce element was used as the background to ensure the validity of the test. The results are shown in Figure 3d. The results show that the content of Cu in the PBS dispersion of Cu/CeO_2_ NPs shows a time-dependent increase trend, reaching the peak at 15 min, with a concentration of 1 mg/mL; it also shows a concentration-dependent increase trend. The content of Ce in the dispersion at the same time did not increase significantly, indicating that the test results only represent the total number of ions in the solution environment. The experimental details are reported in the Cu ion release test section.

To verify the batch-to-batch consistency of the antibacterial performance of the system, we further evaluated the in vitro bactericidal effects of three batches of Cu/CeO_2_ NPs on *MRSA* (Figure S8). At a concentration of 0.5 μg/mL and 1 h co-incubation period, all batches achieved a bactericidal rate of over 99.9% against an initial *MRSA* concentration of 1 × 10^8^ CFU/mL. This highly consistent bactericidal efficacy confirmed the excellent batch-to-batch stability and process reproducibility of the in vitro antibacterial activity of Cu/CeO_2_ NPs.

Based on the multi-characterization results including the hydrated particle size, zeta potential, ABTS radical scavenging capacity, and in vitro antibacterial assay, the three independently prepared batches of Cu/CeO_2_ NPs demonstrated high reproducibility in chemical and biological properties. The above results systematically confirm that this synthetic strategy has excellent process robustness and batch-to-batch equivalence, providing a solid and reliable quality foundation for its transformation and application into engineered antibiotic formulations.

### 2.4. In Vitro Cell Experiments

Figure 4 demonstrates the cellular inflammatory regulation performance of the material. Figure 4a shows that at a drug concentration below 160 μg/mL, both Cu/CeO_2_ NPs and CeO_2_ NPs have no significant cytotoxicity, and the cell compatibility of Cu/CeO_2_ NPs is slightly better than that of CeO_2_ NPs. Live and dead cell staining (Figure 4b) and the corresponding statistical results (Figure 4d) further confirm this conclusion. The ROS staining of cells (Figure 4c) and the corresponding statistical results (Figure 4e) indicate that Cu/CeO_2_ NPs can effectively remove ROS in cells. The expression levels of cell oxidative stress metabolites MDA (Figure 4f), GSH (Figure 4g), and SOD (Figure 4h) show that Cu/CeO_2_ NPs can effectively alleviate the lipid peroxidation state of inflammatory endothelial cells, and its performance is superior to that of CeO_2_ NPs. In terms of cytokine regulation, Cu/CeO_2_ NPs have a stronger inhibitory effect on HIF-1α (Figure 4i), TNF-α (Figure 4j), and IL-1β (Figure 4k) than CeO_2_ NPs, indicating that it has the ability to reshape the microenvironment of inflammatory endothelial cells. Notably, the down-regulation effect of Cu/CeO_2_ on HIF-1α is closely related to its conductive enzyme activity, suggesting that this material can alleviate the hypoxic microenvironment of inflammatory cells. In summary, the results of in vitro antibacterial experiments and cell function evaluations indicate that Cu/CeO_2_ NPs have potential application value in promoting the healing of bacterial infected wounds.

### 2.5. Wound Healing Experiment

Figure 5 illustrates the therapeutic effect of Cu/CeO_2_ NPs on *MRSA*-infected mouse wound models. The hemolysis assay (Figure S5) results indicated that Cu/CeO_2_ NPs did not cause significant hemolysis within a wide concentration range (≤100 μg/mL). Figure 5a shows a schematic diagram of the treatment cycle: starting from 24 h after bacterial infection, the treatment group mice were locally administered 40 μL of a suspension containing 1 μg/mL Cu/CeO_2_ NPs on the wound surface, while the model group and the sham operation group were given the same volume of PBS solution. The administration was continued for 3 consecutive days. The left, middle, and right columns of Figure 5b respectively display representative images of the wounds in each group, the corresponding wound area projection diagrams, and the bacterial statistics of the wounds on the 9th day. The results show that the wound areas of each group began to show significant differences from the 5th day, and the differences further expanded by the 9th day. On the 9th day, the wounds in the model group were still in the scabbing stage, and partial epithelialization was observed in the sham operation group, while the wounds in the treatment group were basically re-epithelialized and close to complete healing. The bacterial statistics of the wounds on the 9th day indicated that the number of colonies in the treatment group was significantly lower than that in the model group and the sham operation group. The wound area statistics of all mouse samples (*n* = 6) in this experiment are shown in Figure 5c, presenting a consistent conclusion. The indicators such as MDA (Figure 5d), GSH (Figure 5e), SOD (Figure 5f), TNF-α (Figure 5g), IL-1β (Figure 5h), and HIF-1α (Figure 5i) showed that Cu/CeO_2_ NPs could significantly alleviate the ROS overload and chronic inflammation in the wound of *MRSA*-infected mice. The biochemical criterion measured the serum from different animals. The expression level of Vascular Endothelial Growth Factor-A (VEGF-A) (Figure 5j) increased successively in the control group, the sham operation group, the *MRSA* infection group, and the *MRSA* group with Cu/CeO_2_ NP intervention, indicating that skin wound formation could initiate the repair response, and *MRSA* infection further intensified this response to cope with inflammatory damage. Meanwhile, Cu/CeO_2_ NP intervention significantly enhanced the expression of VEGF-A, suggesting that the Cu/CeO_2_ NPs could synergistically promote angiogenesis and tissue repair in the wound. At this concentration, the therapeutic effect of Cu/CeO_2_ NPs on the *MRSA*-infected mouse wound model is highly satisfactory. We attribute this to the synergistic effect of its antibacterial activity and its ability to clear ROS and alleviate oxidative stress, thereby inhibiting persistent infection while simultaneously improving the local inflammatory microenvironment. Cu/CeO_2_ NPs were directly served a dispersion, which is fundamentally different from that of the gel dressing system. Therefore, the wound healing efficacy of Cu/CeO_2_ NPs was not directly compared with that of commercial gel dressings in this study. We do not deny that unmodified CeO_2_ NP carriers can promote wound healing by regulating the inflammatory microenvironment alone [34,35]; however, this study focuses more on integrating the pathogen clearance function into the material system and systematically evaluating its synergistic antibacterial and wound repair-promoting capabilities at the wound site.

Figure 6 shows the staining results of skin tissue sections from mice. On the 10th day, the mice were sacrificed and the tissues were sectioned. The hematoxylin–eosin (H&E) and Masson staining results are presented in the upper and lower rows of Figure 6, respectively. The H&E staining results indicated that in the control group, the epidermis of the mice had a clear stratification and regular cell arrangement, with no obvious inflammatory cell infiltration. In the sham operation group, the epidermis was slightly thickened, and the stratum corneum was slightly hyperplastic. The cell polarity was normal, and there were a few scattered inflammatory cells, without significant pathological changes. In the model group, the epidermis was significantly thickened, the cell arrangement was disordered, the collagen fibers were disordered, and the infiltration of inflammatory cells was significantly increased, with obvious activation of fibroblasts, presenting a typical pathological damage state. In the treatment group, the epidermal thickness significantly decreased, the cell arrangement returned to regularity, the collagen fibers were arranged loosely but gradually in an orderly manner, the infiltration of inflammatory cells was significantly reduced, vascular dilation was alleviated, and the activation of fibroblasts was decreased, with a significant improvement in pathological damage. The Masson staining results showed that in the control group, the dermal collagen fibers of the mice were light blue and loosely wavy, with normal collagen deposition. In the sham operation group, the dermal collagen was basically regular, with a slightly darker light blue color and a slight increase in density. In the model group, the dermal collagen layer was dark blue, with a disordered and dense arrangement and obvious abnormal hyperplasia of collagen. In the treatment group, the blue staining of collagen fibers became lighter, the deposition decreased, and the arrangement recovered from disordered to close and orderly, with a significant improvement in skin fibrosis.

## 3. Materials and Methods

Chemicals

Citric acid, Innochem, Beijing, China; copper chloride, Innochem, China; 2,2′-Azino-bis(3-ethylbenzothiazoline-6-sulfonic acid), Aladdin, China; BCA Protein Assay Kit, Solarbio, Beijing, China; Lipopolysaccharide, Titan, Shanghai, China; TNF-α ELISA Kit, HIF-α Assay Kit and IL-1β ELISA Kit, Fankeiwei, Shanghai, China; Glutathione Assay Kit, Malondialdehyde Assay Kit and superoxide Dismutase Assay Kit, Nanjing Jiancheng, Nanjing China; Dulbecco’s Modified Eagle’s Medium, Gibco Thermo Fisher Scientific, Waltham, MA, USA; 4′,6-Diamidino-2-phenylindole, reactive Oxygen Species Assay Kit, calcein/PI Cell Viability and Cytotoxicity Assay Kit, Beyotime, Shanghai, China; Cerium nitrate, 1,1-Diphenyl-2-picrylhydrazyl, ethylenediaminetetraacetic acid, 3,3′,5,5′-Tetramethylbenzidine and ferrous sulfate, Aladdin, Shanghai, China.

Instruments

Laboratory instruments include a zeta potential analyzer manufactured by Brookhaven (Nashua, NH, USA), UV–vis spectrophotometer, X-ray diffractometer (XRD) and X-ray photoelectron spectrometer (XPS) from Shimadzu (Tokyo, Japan), transmission electron microscope (TEM) and inductively coupled plasma spectrometer (ICP) supplied by Thermo Fisher Scientific (Waltham, MA, USA). Biological experimental equipment consists of a biosafety cabinet and CO_2_ incubator produced by Thermo Fisher Scientific (USA), tabletop low-speed centrifuge from Xiangyi Instrument (Xiangtan, China), refrigerated centrifuge of Thermo Fisher Scientific (USA), ultrasonic cell disruptor manufactured by Ningbo Scientz Biotechnology, microplate reader from BioTek (Winooski, VT, USA), and inverted fluorescence microscope made by Nikon (Tokyo, Japan).

Synthesis of CeO_2_ NPs

The synthesis of CeO_2_ NPs followed the method in the reference and was appropriately optimized [27]. Hexahydrate cerium nitrate (Ce(NO_3_)_3_·6H_2_O, 0.44 g, 1.9 mmol) and citric acid (C_6_H_8_O_7_, 0.20 g, 1.0 mmol) were successively dissolved in 4.0 mL of ultrapure water at room temperature. Magnetic stirring was carried out for 0.5 h until complete dissolution, then the solution was transferred to a constant temperature water bath and reacted continuously at 50 °C for 24 h. After the reaction, the precipitate was collected by centrifugation at 10,000× *g* rpm for 10 min. The obtained product was repeatedly washed with ultrapure water three times to remove unreacted precursors and by-products, and finally dried in a vacuum drying box at 60 °C for 12 h to obtain light yellow CeO_2_ NP powder.

Synthesis of Cu/CeO_2_ NPs

The synthesis of Cu/CeO_2_ NPs followed the method in the reference and was appropriately optimized [28]. Take 20 mg of CeO_2_ NPs and disperse them in 25 mL of ultrapure water. Ultrasonic treatment for 10 min ensures uniform dispersion. Add 2, 4, or 8 mL of 1 mg/mL CuCl_2_ aqueous solution to the dispersion system. Stir at room temperature for 0.5 h to ensure uniform copper ions. Transfer the resulting mixture to a quartz reaction vessel with a jacket. Set the circulating water temperature at 10 °C. Then, introduce 0.2 L/min of high-purity hydrogen gas into the dispersion system and turn on a 300 W xenon light source to stir the reaction for 6 h. After the reaction, centrifuge at 10,000× *g* rpm for 10 min to collect the precipitate. The product is repeatedly washed with ultrapure water three times and finally dried in a vacuum drying oven at 60 °C for 12 h to obtain Cu/CeO_2_ NPs.

Enzyme activity and chemical reducibility tests

Prepare the material dispersion solution and substrate solution. In the control group, add a certain volume of ultrapure water to the substrate solution and incubate for a certain period of time. Measure the absorbance at a specific wavelength and record it as A0; in the experimental group, add a certain volume of the material dispersion solution to the substrate solution and incubate for a certain period of time. Measure the absorbance at the corresponding wavelength and record it as Ax. The substrate clearance efficiency is calculated using the following formula:

Clearance rate = (A0 − Ax)/A0

Prepare 1 mg/mL CeO_2_ NPs, B1, B2, and B3 dispersion solutions.

Prepare 98 mM H_2_O_2_ solution (0.1 mL of 30% hydrogen peroxide solution diluted to 10 mL); test the absorbance at 240 nm.

Prepare OH free radical solution (130 μL of 250 μM TMB mixed with 100 μL of 2 mM H_2_O_2_); test the absorbance at 652 nm.

Prepare ABTS free radical solution (7 mM ABTS and 2.45 mM mixed overnight); test the absorbance at 734 nm.

Prepare DPPH free radical solution (1 mg DPPH in 24 mL ethanol); test the absorbance at 519 nm.

Oxygen-free water was prepared by boiling ultrapure water and purging it with Ar gas until it cooled to room temperature. A 1 μg/mL material dispersion was prepared using oxygen-free water, and 20 μL of 98 H_2_O_2_ mM solution was added. Subsequently, the O_2_ concentration was monitored once every 3 min using a dissolved oxygen meter (HJPBJ-608, INESA Instruments, Shanghai, China).

In vitro antibacterial experiment

Place the monoclonal bacteria in liquid culture medium and incubate for 14 h. Collect the bacteria at 8000× *g* rpm for 1 min. Wash the bacteria three times with PBS, and then resuspend them in PBS to form a bacterial solution with an O.D. value of 0.4 (CFU value ≥ 107). Take 0.4 mL of the bacterial solution, mix it with a certain volume of material dispersion solution, and dilute it to 2 mL. After a certain period of reaction, take samples for serial dilution and plate counting to determine the number of colonies B (the control group is recorded as B0, the experimental group as Bx). Calculate the sterilization rate through the following formula:

Clearance rate = (B0 − Bx)/B0 (The sample size n of all experiments is ≥3.)

Bacterial morphology experiment

Take a certain volume of bacterial dispersion solution and drop it onto a silicon wafer. Let it air dry naturally, then immerse it in a 2.5% acetaldehyde solution overnight. Subsequently, immerse it in 20%, 40%, 60%, 80%, and 100% ethanol water solutions for 10 min each, and finally let it air dry before conducting SEM testing.

Cu^2+^ release experiment

Take 50 mg of Cu/CeO_2_ NPs dispersed in 50 mL of PBS solution. After ultrasonic dispersion is completed, incubate it in a shaking incubator at 37 °C (180 rpm). At fixed time points, take 10 mL of the dispersion solution and centrifuge it (10,000 rpm/10 min) to collect the supernatant, which will be used for ICP testing.

Animal experiments

ICR mice (male, 6–8-week old, 25–30 g) were obtained from Beijing Vital River Laboratory Animal Technology, and were adaptively fed for 7 days before being used for the experiment. Thirty-two mice were randomly divided into 4 groups (n = 8/group): normal control group, sham operation group, model group, and treatment group. After 7 days of adaptive feeding, they were anesthetized with isoflurane inhalation; mice in the sham operation group, model group, and treatment group had circular full-thickness skin wounds with a diameter of 0.8 mm made at the center of the back. Subsequently, the wounds in the model group and treatment group were locally dripped with 40 μL of *Staphylococcus aureus* suspension (O.D.600 = 1.0), while the sham operation group and normal control group were dripped with the same volume of PBS solution. Then, 24 h after infection, the treatment group was given 40 μL of the experimental material dispersion solution (concentration 1 μg/mL), while the model group and sham operation group were given the same volume of PBS solution. The administration lasted for 3 days, with an interval of 24 h each time. On the 10th day after the operation, the animals were sacrificed, and the blood from the posterior orbital venous plexus was collected for serum biochemical index detection. The wound tissues were embedded in paraffin and stained with HE and Masson.

Cell viability

HUVECs were seeded at a density of 13 × 10^4^ cells/mL in 96-well plates. Each group was set up with 6 duplicate wells, and a blank control group was also included. The plates were placed in a cell culture incubator for 24 h of culture. After removing the culture medium from each well, LPS was added for 24 h of treatment, and different concentrations of materials were co-incubated at 37 °C for 24 h. Subsequently, 10 μL of CCK-8 solution was added to each well for a further 4 h co-incubation, and the OD values at 450 nm in each well were measured in a microplate reader.

Cell ROS and Cell Viability Staining

HUVECs were seeded at a density of 12 × 10^4^ cells/mL in 24-well plates and cultured in a cell incubator for 24 h. When the cell confluence reached 80–90%, the cells were first treated with LPS for 24 h, and then treated with different concentrations of the materials for another 24 h. After removing the culture medium, the cells were washed three times with PBS.

For ROS staining, add DCFH-DA (15 μM) and incubate for 30 min; for viability staining, add 2 μL Calcein-AM and incubate for 20 min. Subsequently, remove the culture medium and wash the cells three times with PBS, add 5 μL of DAPI (5 μg/mL), and incubate for 30 min. Remove the culture medium and add 1 mL of basic medium to each well to maintain cell viability. Collect the cell fluorescence images using an inverted fluorescence microscope.

Biochemical Indicators Detection

HUVECs that had grown well were inoculated into 100 mm culture dishes. When the cells reached 80–90% confluence at the bottom of the dish, they were treated with LPS for 24 h. Then, they were treated with different concentrations of the materials for another 24 h. Subsequently, the cells were collected for cell lysis, and their concentrations were measured. Then, the levels of relevant indicators within the cells were detected using kits such as MDA, GSH, SOD, TNF-α, IL-1β, and HIF-1α.

Statistical analysis

Independent sample *t*-test was used for comparison between two groups; one-way ANOVA was employed for three or more groups. When significant results were obtained, post hoc pairwise comparisons were conducted using the Tukey method. Significance levels: * *p* < 0.05, ** *p* < 0.01, *** *p* < 0.001, **** *p* < 0.0001.

All animal procedures were approved by the Animal Care and Welfare Committee of Guizhou Medical University (Approval No. SYXK2025-0001).

## 4. Conclusions

This study designed and engineered Cu/CeO_2_ NPs that can spontaneously exert dual antibacterial and anti-inflammatory functions under physiological conditions without any external stimuli, demonstrating significant potential as a novel nano-antibiotic. Chemical reduction experiments showed that the introduction of Cu did not weaken the inherent CAT and overall antioxidant capacity of CeO_2_ NPs. At a very low concentration of 0.5 μg/mL, Cu/CeO_2_ NPs could efficiently kill *MRSA* and *E. coli*. Meanwhile, Cu/CeO_2_ NPs prepared from different batches exhibit consistent chemical reduction activity and in vitro antibacterial performance, confirming their suitability for practical engineering applications. Mechanism studies revealed that Cu/CeO_2_ NPs mainly killed bacteria by continuously releasing Cu^2+^ from Cu species in physiological environments, thereby inducing the loss of bacterial cell membrane integrity. Cell experiments demonstrated that Cu/CeO_2_ NPs could significantly clear intracellular ROS and bidirectionally regulate the expression levels of key inflammatory factors. In a full-thickness skin wound model infected with *MRSA*, Cu/CeO_2_ NPs could significantly promote the nearly complete healing of a standardized circular wound with an initial diameter of 8 mm within 10 days at a local administration concentration as low as 1 μg/mL under physiological conditions without any external stimuli such as light, heat, or pH regulation. The Cu/CeO_2_ NPs reported in this study achieve time-dependent antibacterial and anti-inflammatory effects at extremely low working concentrations without any external stimuli, showing great potential as a nano-antibiotic to replace traditional antibiotics. This engineered nano-antibacterial agent not only provides a feasible solution to the global public health challenge of drug-resistant bacterial infections, but also points out a more targeted development direction for the design concept and structure–activity relationship research of nano-antibiotics.

## Data Availability

Data supporting the reported results are contained within the article or are available from the corresponding authors.

## Supplementary Materials

The following supporting information can be downloaded at https://www.mdpi.com/article/10.3390/molecules31111957/s1. Figure S1: The Cu/Ce mass ratio for different batches; Figure S2: The hydrodynamic size of Cu/CeO_2_ NPs; Figure S3: The zeta potential of Cu/CeO_2_ NPs and CeO_2_ NPs; Figure S4: The hydrodynamic size and zeta potential of different batches of Cu/CeO_2_ NPs; Figure S5: CAT of CeO_2_ NPs, B1, B2, and B3; Figure S6: The ABTS eliminate ratio of different batches of Cu/CeO_2_ NPs; Figure S7: The XRD pattern of CeO_2_ NPs and Cu/CeO_2_ NPs aged in air; Figure S8: The whole XPS spectra of Cu/CeO_2_ NPs; Figure S9: The POD and SOD-like enzyme activity of Batch 2 Cu/CeO_2_ NPs with different concentrations; Figure S10: Representative images of *MRSA* cultures intervened via different batches of Cu/CeO_2_ NPs; Figure S11: The hemolysis assay of Cu/CeO_2_ NPs with different concentrations.
