# Supplementary material for: Engineering Nano-Antibiotics for Accelerating Wound Healing in Drug-Resistant Bacterial Infections
Source: Molecules. 2026 Jun 4;31(11):1957. doi: 10.3390/molecules31111957 (PMC13257607; doi:10.3390/molecules31111957)
Supplement: Supplementary file 1 [file molecules-31-01957-s001.zip › molecules-4293961-supplementary.pdf]

Supporting Information

Engineering Nano-antibiotics for Accelerating Wound Healing in Drug-Resistant Bacterial Infections

Wenmin Yan, Zihao Shen, Shilan liang, Chaozhong Li, Guangwei Feng*, Jinming Zhu*, Jian Feng*

Content

Figure S1 The Cu/Ce mass ratio for different batches.

Figure S2 The hydrodynamic size of Cu/CeO_2 NPs.

Figure S3 The zeta potential of Cu/CeO_2 NPs and CeO_2 NPs.

Figure S4 The hydrodynamic size and zeta potential of different batches of Cu/CeO_2 NPs.

Figure S5 CAT activity of CeO_2 NPs, B1, B2 and B3

Figure S6 The ABTS eliminate ratio of different batches of Cu/CeO_2 NPs.

Figure S7 The XRD pattern of CeO_2 NPs and Cu/CeO_2 NPs aged in air.

Figure S8 The whole XPS spectra of Cu/CeO_2 NPs.

Figure S9 The POD and SOD-like enzyme activity of Batch 2 Cu/CeO_2 NPs with different concentrations

Figure S10 Representative images cultures of MRSA intervened via different batches of Cu/CeO_2 NPs.

Figure S11 The Hemolysis assay of Cu/CeO_2 NPs with different concentrations.

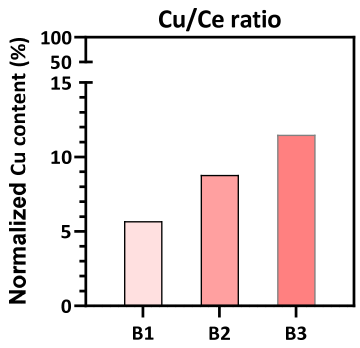

Figure S1 The mass ratio of copper to cerium in batches 1/2/3. The results show that as the proportion of copper precursor feed increases, the elemental content of copper in each batch becomes higher.

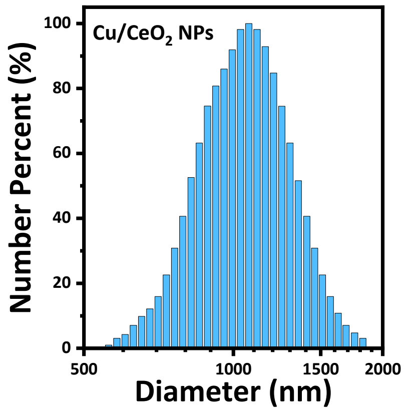

Figure S2 The hydrated size of Cu/CeO_2 NPs was determined, and the results showed that the water and size of the material were approximately 1100 nm.

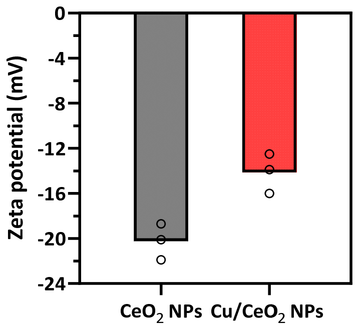

Figure S3 Comparison of the zeta potential of Cu/CeO_2 NPs and CeO_2 NPs. Cu/CeO_2 NPs have a higher zeta potential, indicating that after the doping with Cu, the negative charge of CeO_2 has been weakened. This result is consistent with the results detected by XPS for Cu^+ and Cu^{2+}.

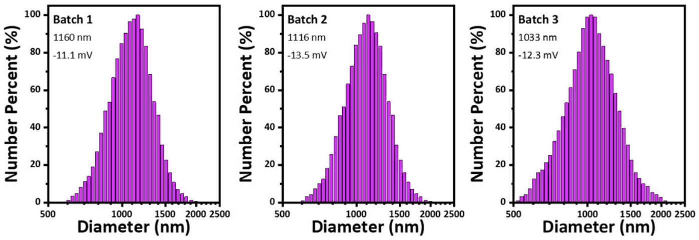

Figure S4 The hydrated size and zeta potential of different batches of Cu/CeO_2 NPs, aiming on the synthesis and property reproducibility. The average hydrated size is 1103 ± 64 nm, with a Relative Standard Deviation (RSD) of 5.9%, while the average zeta potential is -12.3 ± 1.2 mV, with a RSD of 9.8%. This result indicates that the synthesis process has good reproducibility.

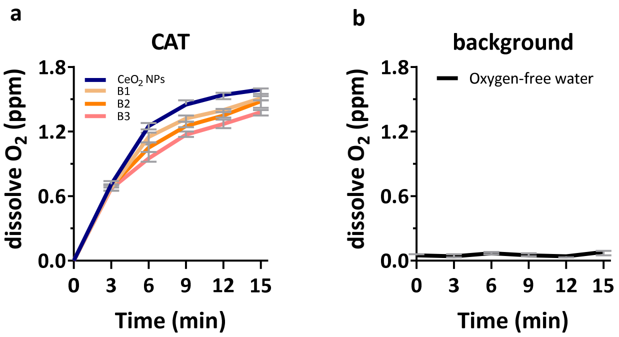

Figure S5 (a) CAT activity of CeO_2 NPs, B1, B2 and B3. (b) Content of dissolved O_2 in oxygen-free water. The dissolved O_2 measured via dissolved oxygen meter.

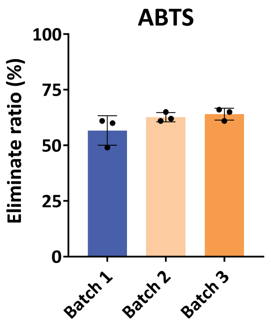

Figure S6 The ABTS eliminate ratio of different batches of Cu/CeO_2 NPs, aiming on the synthesis and property reproducibility. The ABTS radical scavenging assay was performed on different batches of Cu/CeO_2 NPs at a concentration of 1 μg/mL, with an initial absorbance of approximately 1.5 at 732 nm and co-incubation time for 10 min. The experimental conditions were consistent with those reported in Figure 2.

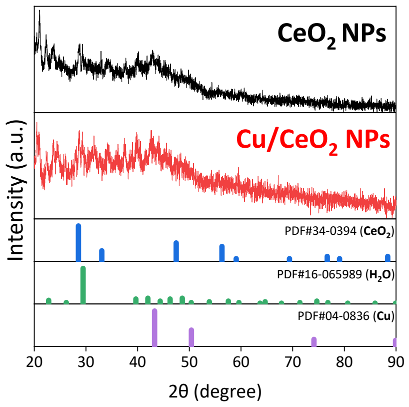

Figure S7 XRD pattern of CeO_2 NPs and Cu/CeO_2 NPs aged in air. The broadening of the diffraction peaks can be attributed to the introduction of a large amount of crystalline water, which directly leads to the material's dynamic size in the aqueous phase environment being significantly larger than the dry state size measured by TEM.

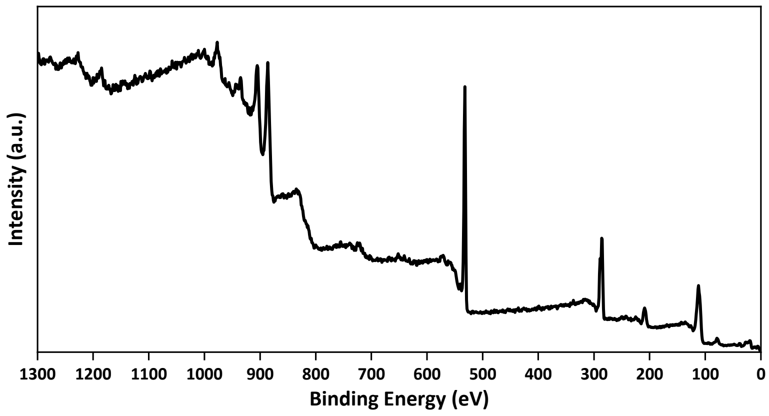

Figure S8 The whole XPS spectra of Cu/CeO_2 NPs.

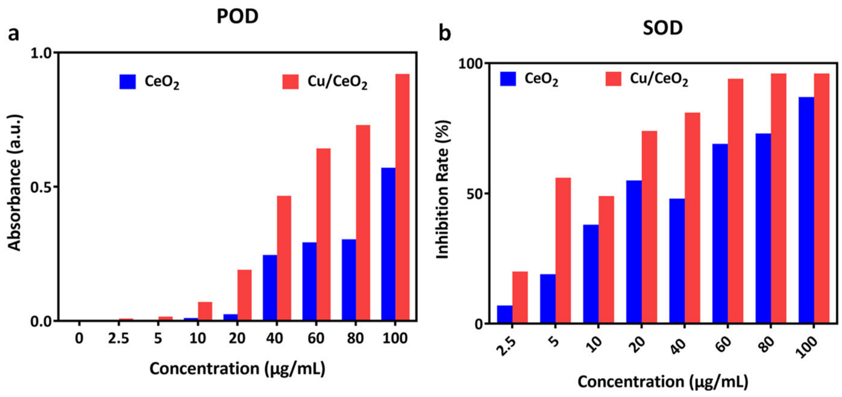

Figure S9 The concentration independence POD (a) and SOD (b) enzyme activity of B2 Cu/CeO_2 NPs. The results show that the Cu/CeO_2 NPs. exhibits significantly superior POD and SOD enzyme activities compared to the CeO_2 NPs., and has the potential to trigger a cascade of enzyme activities in the disease microenvironment to produce O_2.

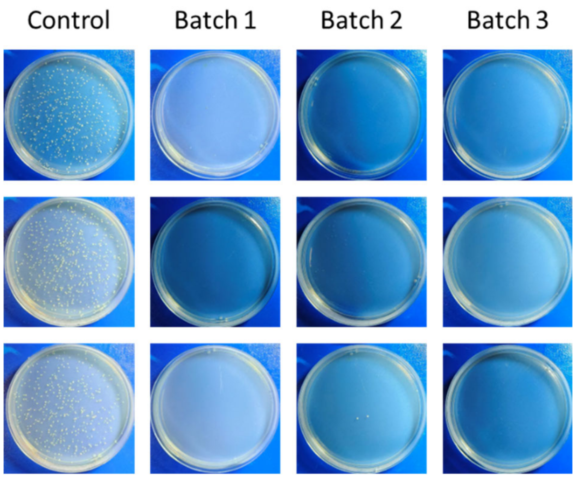

Figure S10 Representative images cultures of MRSA intervened via different batches of Cu/CeO2 NPs. The CFU of MRSA was approximately 1x10^8, with the concentration of Cu/CeO_2 NPs at 0.5 μg/mL and a co-incubation time of 1 h.

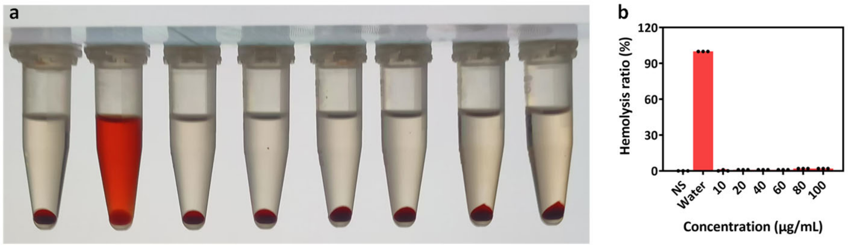

Figure S11 The hemolysis assay of Cu/CeO_2 NPs. The results showed that the material did not cause hemolysis at working concentrations (≤ 100 μg/mL), which was much higher than the actual usage concentration in the animal experiments in this study (1 μg/mL).
